# A Sub-Aperture Overlapping Imaging Method for Circular Synthetic Aperture Radar Carried by a Small Rotor Unmanned Aerial Vehicle

**DOI:** 10.3390/s23187849

**Published:** 2023-09-13

**Authors:** Lina Chu, Yanheng Ma, Bingxuan Li, Xiaoze Hou, Yuanping Shi, Wei Li

**Affiliations:** 1 Department of UAV Engineering, Shijiazhuang Campus, Army Engineering University, Shijiazhuang 050003, China; chulina@aeu.edu.cn (L.C.); libingxuan2021@aeu.edu.cn (B.L.); 1472583699@163.com (X.H.); 1102061@sjzc.edu.cn (Y.S.); liweiweitnt@126.com (W.L.); 2 College of Mechanical and Electrical Engineering, Shijiazhuang University, Shijiazhuang 050035, China

**Keywords:** circular synthetic aperture radar (CSAR), adaptive overlapping sub-aperture, Back Projection (BP), small rotor unmanned aerial vehicle (SRUAV), correlation coefficient, coefficient of variation

## Abstract

Circular synthetic aperture radar (CSAR) can obtain higher image resolution and more target information using 360° observation of the target. Due to the anisotropy of target scattering characteristics in the actual scene, the sub-aperture imaging method is usually used for CSAR imaging. However, the uniformly divided overlapping sub-aperture CSAR imaging algorithm only considers phase compensation, ignoring the effect of target scattering characteristics on echo amplitude. In CSAR imaging scenarios carried by small rotor unmanned aerial vehicles (SRUAVs), the size of the observed scene cannot be ignored compared to the distance between the target and the antenna and the effect of the anisotropy of the target scattered energy on the echo amplitude should be considered. In this paper, a sub-aperture CSAR imaging method based on adaptive overlapping sub-aperture is proposed. First, the boundary points of the sub-aperture are determined by analyzing the correlation coefficient and the variation coefficient of the energy function. Next, the overlapping sub-aperture division schemes are automatically generated by screening and combining the boundary points. The sub-aperture images are then generated by a Back Projection (BP) algorithm. Finally, sub-aperture image registration and incoherent superposition are used to generate the final CSAR image. Verified by the CSAR field echo data, the proposed method can realize imaging of the original echo data without the Inertial Navigation System (INS) and Global Positioning System (GPS) observation data. Compared with the CSAR full-aperture BP imaging algorithm, the entropy of the image generated by the proposed method increased by 66.77%. Compared with the sub-aperture CSAR imaging algorithm, the entropy of the image generated by the proposed method was improved by 11.12%, retaining more details of the target, improving the target contour features, and enhancing the focusing effect.

## 1. Introduction

Circular synthetic aperture radar (CSAR) realizes 360° omnidirectional observation of a target by controlling the radar antenna to move along the circular trajectory, providing higher image resolution, more target information, and even three-dimensional imaging capability compared with Linear SAR (LSAR) [1,2,3,4]. Since Soumekh first proposed the CSAR imaging mode in 1996 [5], it has been of great interest to the French Aerospace Lab (ONERA) [4,5,6,7], Swedish Defence Research Agency (FOI) [4,8], American Air Force Research Laboratory (AFRL) [9,10,11,12,13,14], German Aerospace Center (DLR) [15,16,17], and other foreign institutions such as the National University of Defense Technology [18,19,20], Institute of Electronics, and Chinese Academy of Sciences [21], have successfully conducted CSAR outfield airborne flight experiments and verified the imaging capability of CSAR imaging modes by processing the measured data.

CSAR 2D imaging algorithms are divided into two categories. The first is time-domain imaging algorithms, represented by the Back Projection (BP) and Fast Factorized Back Projection (FFBP) algorithms [22]. The second is frequency domain imaging algorithms, represented by the wavefront reconstruction imaging method [23] and the frequency domain imaging algorithm based on sub-aperture [24]. The wavefront reconstruction algorithm uses a fast Fourier transform (FFT) to improve the operation efficiency, but the inversion of the system kernel function matrix increases the complexity of this algorithm [5]. The premise of the frequency domain imaging algorithm is the far-field hypothesis [25], which is not fully satisfied by the CSAR carried by small rotorcraft UAVs (SRUAVs). The BP algorithm can realize arbitrary trajectory imaging and high-precision scene imaging, and it is the most commonly used CSAR imaging algorithm at present [26]. However, the BP algorithm requires a large amount of computation and has low imaging efficiency. The FFBP algorithm can significantly reduce the burden in a recursive way, maintain the accuracy and applicability of the BP algorithm, and optimize the running speed via parallel processing hardware, affording it broad application prospects in the field of CSAR imaging [27,28,29]. However, each level of sub-aperture merging in the FFBP algorithm corresponds to an update of the image coordinate system, which increases the difficulty of image accumulation.

The above imaging methods process full-aperture data without considering the anisotropy of target scattering characteristics in the actual scene, resulting in a low signal-to-noise ratio (SNR) in the CSAR images and the loss of scattering information [30,31]. In the measured scene, the scattering characteristics of most targets are only constant within a small angle; that is, the scattering energy of targets is only distributed within part of the aperture [32]. The sub-aperture CSAR imaging method can solve the anisotropy problem of target scattering characteristics. The CSAR full-aperture echo data are divided into sub-aperture data according to the azimuth rotation angle, and the sub-aperture data are, respectively, coherently imaged, with the sub-aperture images being incoherently superimposed to obtain the final CSAR images [30,33]. The sub-aperture CSAR imaging method based on sub-aperture division and incoherent superposition does not consider the difference in scattering energy of anisotropic targets, resulting in low signal-to-noise ratio and false scattering points. The image quality of CSAR can be effectively improved by dividing the echo data into overlapping sub-apertures [34]. However, the uniform division of overlapping sub-apertures only considers phase compensation and ignores the influence of target scattering characteristics on echo amplitude. Especially when the size of the observed scene is not negligible compared with the distance between the target and the antenna, the variation in echo amplitude created using the anisotropy of the target scattering energy causes the strong energy sidelobe to submerge the weak energy main lobe in the image, and the signal-to-noise ratio of the image is low, leading to a serious defocus.

To address the above sub-aperture CSAR imaging problems, this paper proposes a CSAR imaging method based on adaptive overlapping sub-aperture. First, following analysis of the raw data correlation coefficient and energy function variation coefficient, the boundary points of the sub-aperture are set at the position that is least likely to be the center of strong scattering energy; this ensures that the strong scattering energy is completely located in one sub-aperture and improves the signal-to-noise ratio of the image. To maximize the image resolution of each sub-aperture, the overlapping sub-aperture partition schemes are then automatically generated by screening and combining the sub-aperture boundary points to ensure that the sub-aperture width is as close as possible to the theoretical maximum sub-aperture width. Finally, the BP algorithm is used to generate the sub-aperture images, and the final CSAR image is generated by registration fusion. The imaging processing results for the CSAR echo data carried out by the SRUAV verify the effectiveness of the method presented in this paper.

This paper is organized as follows. Section 2 introduces the CSAR imaging geometry, sub-aperture imaging algorithms, and the limitations of equipartition overlapping sub-aperture. Next, Section 3 describes the process and key technologies for the CSAR imaging method based on adaptive overlapping sub-aperture. In Section 4, the results and analysis of the proposed method on the X-band CSAR echo dataset carried by an SRUAV are presented and discussed. Finally, in Section 5, conclusions are drawn.

## 2. CSAR 2D Imaging Method Based on Sub-Aperture

### 2.1. Geometry of CSAR

**Figure 1:**
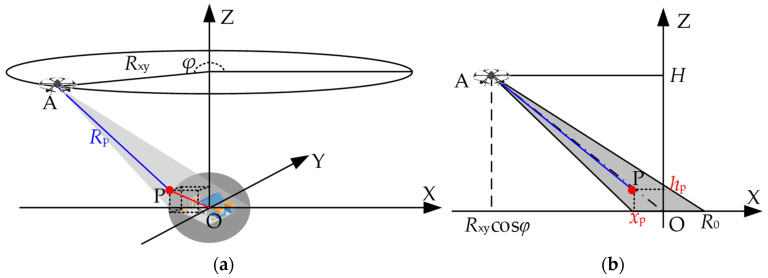
CSAR imaging geometric model by an SRUAV; (**a**) CSAR imaging geometry diagram; (**b**) side view.

The geometry of a CSAR carried by an SRUAV is shown in Figure 1. The SRUAV with the CSAR flies around the observation scene at a fixed altitude H in a circle. The center of the antenna beam always points at the center of the imaging scene, collecting echoes of the scene with a ground radius of $R_{0}$ [35]. During the movement of the CSAR platform, the instantaneous slant range between the platform and the target P at any point can be expressed as

(1) $$R_{p}\left(\varphi \right)=\sqrt{{\left(R_{\mathrm{xy}}\cos\varphi -x_{p}\right)}^{2}+{\left(R_{\mathrm{xy}}\sin\varphi -y_{p}\right)}^{2}+{\left(H-z_{p}\right)}^{2}}\begin{array}{cc} & ,\varphi \in \left[0,2\pi \right) \end{array}$$

where $\left(R_{\mathrm{xy}}\cos\varphi ,R_{\mathrm{xy}}\sin\varphi ,H\right)$ is the coordinates of the antenna phase center (APC) of point A; $\varphi \in \left[0,2\pi \right)$ is the rotation angle with the positive of the *X*-axis as the reference direction; $\left(x_{p},y_{p},z_{p}\right)$ is the position coordinate of any point target P in the scene.

### 2.2. BP Algorithm

Assuming that the SAR system transmits a frequency-modulated continuous wave (FMCW) signal, the range-compressed signal can be expressed as follows [36]:

(2) $$s_{r}\left(\tau ,\varphi \right)=\sigma_{p}\cdot \sin c\left\{B\left[\tau -\frac{2R_{p}\left(\varphi \right)}{c}\right]\right\}\cdot \exp\left[-j\frac{4\pi f_{c}}{c}\cdot R_{p}\left(\varphi \right)-j\frac{4\pi K_{r}}{c^{2}}\cdot R_{p}{\left(\varphi \right)}^{2}\right]$$

where τ is the fast time; $\sigma_{p}$ denotes the scattering coefficient of target point P; B is the bandwidth of the transmitted signal; c denotes the propagation velocity of the electromagnetic wave; $f_{c}$ denotes the center frequency of the transmitted signal; $K_{r}$ is the chirp-rate; and $\sin c\left(\cdot \right)$ is the sinc function, which can be expressed as

(3) $$\sin c\left(x\right)=\frac{\sin\left(\pi x\right)}{\pi x}$$

The principle of the BP imaging algorithm is as follows: first, the instantaneous slant range of each pixel in the preset grid is calculated; the phase compensation of echo is then carried out pixel by pixel along the azimuth direction; finally, coherent superposition is performed to obtain a two-dimensional image [37]. Let $\left(x_{p},y_{p},0\right)$ be any point on the ground flat grid (two-dimensional imaging $z_{p}=0$); the echo signal after distance compression is coherently superimposed after phase delay compensation along the rotation angle. The imaging results for this point can be expressed as

(4) $$I\left(x_{p},y_{p}\right)=\int_{0}^{2\pi }s_{r}\left(\tau ,\varphi \right)\cdot \exp\left[j\frac{4\pi f_{c}}{c}\cdot R_{p}\left(\varphi \right)+j\frac{4\pi K_{r}}{c^{2}}\cdot R_{p}{\left(\varphi \right)}^{2}\right]d\varphi$$

where $I\left(x_{p},y_{p}\right)$ is the imaging result of the point $\left(x_{p},y_{p},0\right)$. The segmentation of the imaging grid should be determined according to the resolution of the CSAR system and the imaging requirements.

The BP imaging algorithm is CSAR full aperture coherent superposition, which is suitable for ideal point target imaging with isotropic scattering characteristics. In the actual scene, the scattering characteristics of the target change with the observation angle and are only constant within a small angle, leading to the development of the sub-aperture CSAR imaging algorithm.

### 2.3. Sub-Aperture CSAR Imaging Algorithm

Different from the BP imaging algorithm, the sub-aperture CSAR imaging algorithm evenly divides the full-aperture CSAR echo data and then creates the sub-aperture echo to obtain the sub-aperture image. Finally, it performs coherent or incoherent fusion processing on the sub-aperture image according to the characteristics of the target to obtain the final CSAR image [38].

**Figure 2:**
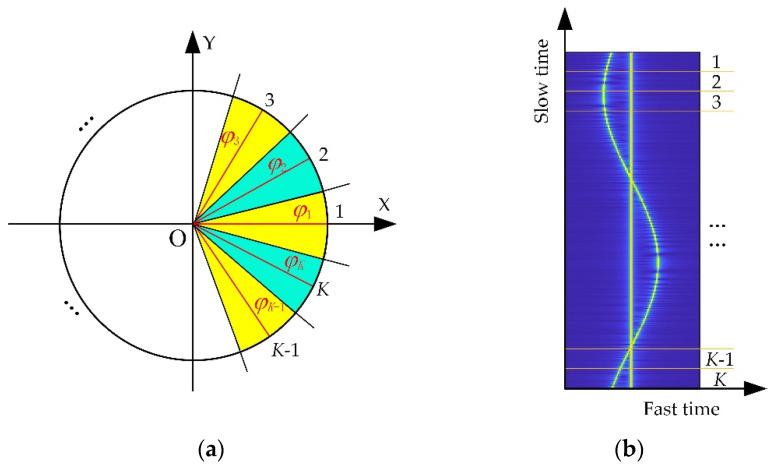
CSAR sub-aperture division diagram; (**a**) division of circular trajectory; (**b**) division of echo data.

As shown in Figure 1, the motion trajectory of the antenna in CSAR mode is a circle, the full aperture angle is Θ=2π, and the synthetic aperture length is $l=2\pi R_{\mathrm{xy}}$. If the full-aperture echo is evenly divided into K sub-apertures, as shown in Figure 2, then the angle of each sub-aperture is φsub=ΘK, the length of the sub-aperture is lsub=lK, and the angle of the k(k=1,2,⋯,K) sub-aperture center relative to the *X*-axis is $\varphi_{k}=\varphi_{\mathrm{sub}}\left(k-1\right)$.

The principle of the sub-aperture CSAR imaging algorithm is as follows. First, the echo data are divided into sub-apertures, and an image of each sub-aperture is then created using the BP algorithm. If the scattering characteristics of the imaging target are isotropic, the images of each sub-aperture are coherently superimposed to obtain high-resolution images. If the scattering characteristics of the imaging target are anisotropic, the images of each sub-aperture are incoherently superimposed to obtain the full-aperture CSAR image.

On the one hand, the sub-aperture CSAR imaging algorithm can improve the efficiency of the BP imaging algorithm using a GPU parallel operation, while on the other hand, it can initially solve the anisotropy of target scattering characteristics; there are, however, several limitations, as described below.

### 2.4. Limitations of Uniformly Overlapping Sub-Apertures

Assuming that the noise in the echo signal is Gaussian white noise, the SNR in the CSAR imaging result is approximated as

(5) $$\mathrm{SNR}=\frac{\sum_{\varphi =\varphi_{s}}^{\varphi_{e}}{\left|\mathrm{abs}\left(s_{r}\left(\varphi \right)\right)\right|}^{2}}{N_{\varphi_{e}}{}_{-\varphi_{s}}}$$

where $\mathrm{abs}\left(\cdot \right)$ denotes the modulus of the complex numbers; $s_{r}\left(\varphi \right)$ represents the echo vector corresponding to the rotation angle φ; $\varphi_{s}$ and $\varphi_{e}$ represent the starting rotation and ending rotation angles of the azimuth coherence accumulation, respectively; and $N_{\varphi_{e}}{}_{-\varphi_{s}}$ is the number of slow-time sampling points corresponding to the coherence accumulation.

When the scattering characteristics of the observed target are isotropic, the amplitude of the radar cross section (RCS) remains unchanged, that is, ${\left|\mathrm{abs}\left(s_{r}\left(\varphi \right)\right)\right|}^{2}$ is a constant. Clearly, it will rise linearly with the increase in the rotation angle. When imaging CSAR echo data from isotropic targets with scattering characteristics, the image azimuth resolution increases with the increase in azimuth rotation angle. Ideally, the resolution of CSAR can reach λ/4 [6], where λ is the wavelength.

**Figure 3:**
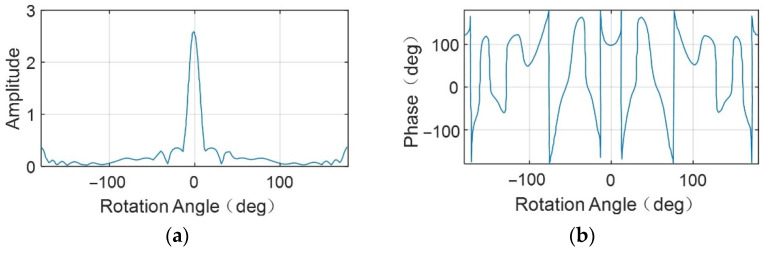
RCS amplitude and phase diagrams of a dihedral angle reflector; (**a**) amplitude; (**b**) phase.

When the scattering energy anisotropy of the target is observed, taking the dihedral angle scatterer as an example, its RCS amplitude and phase change with the azimuth rotation angle, as shown in Figure 3. When the rotation angle is 0°, the RCS amplitude value is the largest, the corresponding phase value is about 100°, and the phase changes slowly. Figure 3 shows that the scattering energy of the dihedral angle scatterer is concentrated around the rotation angle 0°, and coherent accumulation occurs in this rotation angle range. With the increase in rotation angle, the CSAR image resolution increases, and the SNR increases. When the rotation angle exceeds the above range, the RCS amplitude is approximately 0; that is, the energy of the corresponding echo signal is 0. With the increase in the rotation angle, the SNR of the CSAR image decreases, the resolution deteriorates, and there are phenomena such as the main lobe broadening and sidelobe drowning the main lobe [28]. Different from targets with isotropic scattering characteristics, the scattering energy in the echo of targets with anisotropic scattering characteristics is concentrated in the partial molecular aperture [32]. The SNR of CSAR images no longer increases with the increase in rotation angle and even decreases, and the changes are related to the scattering characteristics and geometric characteristics of the observed targets [28].

**Figure 4:**
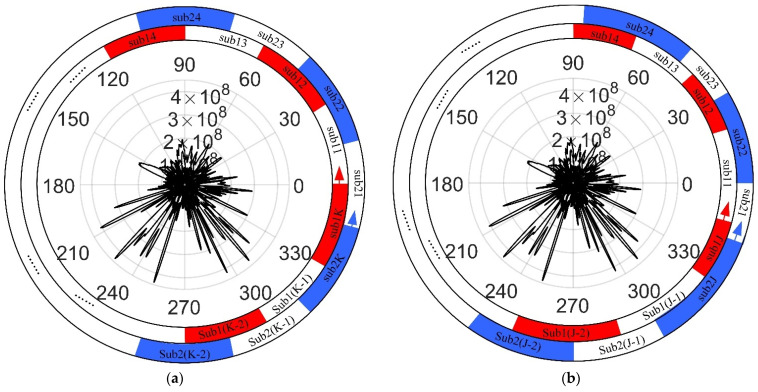
Schematic diagram of the sub-aperture partitioning method; (**a**) traditional method; (**b**) the proposed method.

The full aperture data from CSAR can be divided into fixed sub-aperture centers and equal-width sub-apertures, which can effectively control the deterioration and improve the imaging quality of targets with strong scattering energy [29]. However, direct incoherent fusion of non-overlapping sub-apertures results in missing partial scattering information [28], and the full range of scattering information for the target cannot be obtained. An effective method of solving the above problems is to uniformly overlap sub-aperture CSAR imaging, as shown in Figure 4. First, according to the rotation angle, the full-aperture data are evenly divided into K sub-apertures $sub_{11},sub_{12},\cdots ,sub_{1K}$. Next, with the center of the above sub-apertures as the boundary, the full-aperture data are again evenly divided into K sub-apertures $sub_{21},sub_{22},\cdots ,sub_{2K}$. These sub-apertures overlap each other, which can improve the imaging quality of full-aperture CSAR to a certain extent.

The uniform overlapping sub-aperture method only considers the phase compensation and neglects the effect of target scattering characteristics on echo amplitude. As shown in the central part of Figure 4a, the target scattered energy of the actual scene changes with the change of rotation angle, and the amplitude of energy change is usually across orders of magnitude. When the size of the observation scene is not negligible compared with the distance between the target and the antenna, the anisotropy of the target scattering energy on the echo amplitude cannot be ignored. The uniform division of the overlapping sub-aperture method may divide the same strong scattering energy into different sub-apertures or divide the strong scattering energy and weak scattering energy into the same sub-aperture. As a result, the strong energy sidelobe overwhelms the weak energy main lobe in the image, the image signal-to-noise ratio is low, and the defocus is serious.

## 3. Adaptive Overlapping Sub-Aperture CSAR Imaging Algorithm

**Figure 5:**
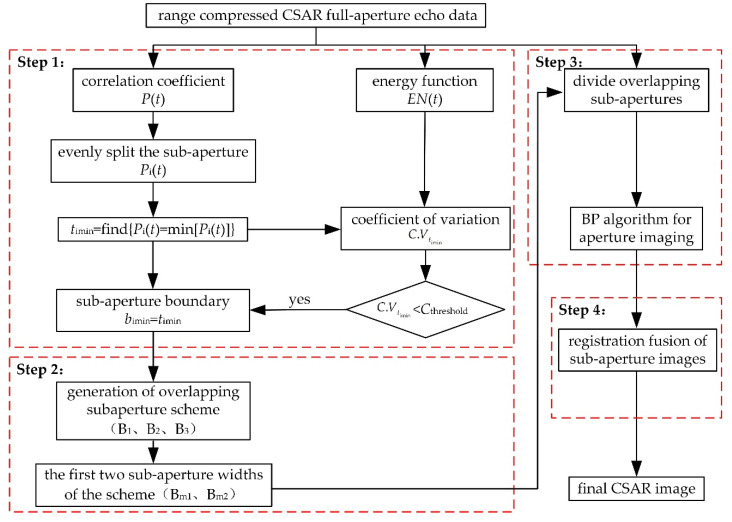
Flowchart of the proposed method.

To solve the above problems in CSAR imaging with uniform overlapping sub-apertures, an adaptive overlapping sub-aperture CSAR imaging method is proposed. This takes the correlation coefficient of echo and the variation coefficient of energy function as the constraint conditions of sub-aperture division to ensure that the sub-aperture is slightly wider than the strong scattering energy and does not truncate the strong scattering energy. The method can be divided into four steps, namely determining sub-aperture boundary points, automatically generating overlapping sub-aperture division schemes, sub-aperture BP imaging, and sub-aperture image registration fusion, as shown in Figure 5. The process and key technologies for each step are explained below.

### 3.1. Determine the Boundary Points of the Sub-Apertures

In Step 1.1, the correlation coefficient and energy function of full-aperture echo data are calculated.

The correlation coefficient represents the strength of the statistical relationship between two variables [39]; the expression is as follows:

(6) $$P\left(t\right)=\mathrm{Mean}\left[P\left(t,t-1\right),P\left(t,t+1\right)\right]$$

where $P\left(t\right)$ represents the correlation coefficient of the echo vector after range compression at the slow time t. $P\left(t,t-1\right)$ represents the correlation coefficient of the echo vector after range compression at slow time t and time t−1, and is expressed as follows:

(7) $$P\left(t,t-1\right)=\left\{\begin{array}{ccc} \frac{C\left[s_{r}\left(wt\right),s_{r}\left(w\left(t-1\right)\right)\right]}{D\left[s_{r}\left(wt\right),s_{r}\left(w\left(t-1\right)\right)\right]} & , & t=2,3,\cdots ,N\\ 0 & , & t=1 \end{array}\right.$$

where $C\left[\cdot ,\cdot \right]$ represents the covariance of two vectors; $D\left[\cdot ,\cdot \right]$ represents the variance of two vectors; $s_{r}\left(wt\right)$ and $s_{r}\left(w\left(t-1\right)\right)$, respectively, represent the echo vector after range compression at slow time t and t−1; w represents the angular rate of circular motion of the antenna, wN=2π; and N represents the slow time sampling number of the CSAR full aperture echo. Since φ=wt and t=1,2,⋯,N, that is, the slow-time sampling along the CSAR trajectory corresponds to the rotation angle, the slow-time sampling along the CSAR trajectory and the rotation angle are no longer distinguished.

The energy function of the CSAR raw data directly reflects the influence of the anisotropy of scattering energy of the actual target on the echo amplitude, which is expressed as follows:

(8) $$EN\left(t\right)=\sum_{n=1}^{N}\left[\mathrm{real}{\left(s_{r}\left(t\right)\right)}^{2}+\mathrm{imag}{\left(s_{r}\left(t\right)\right)}^{2}\right]$$

where $\mathrm{real}\left(\cdot \right)$ and $\mathrm{imag}\left(\cdot \right)$ represent the real and imaginary parts of the echo signal $s_{r}\left(t\right)$, respectively.

**Figure 6:**
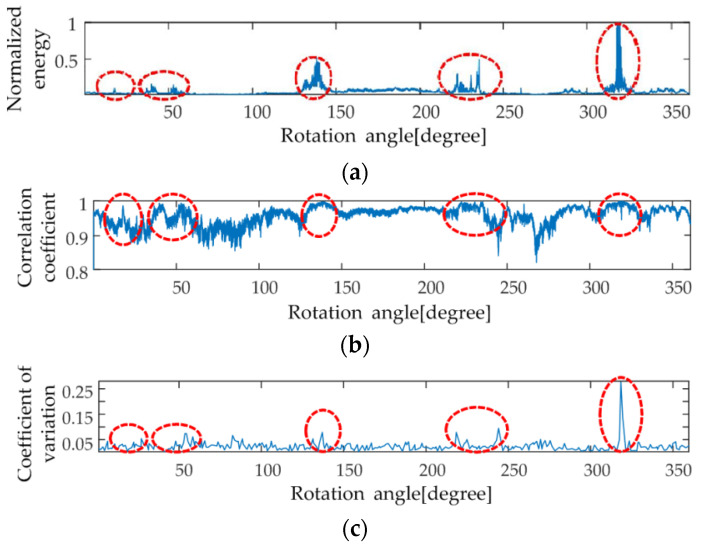
Energy, correlation coefficient, and coefficient of variation distribution; (**a**) energy distribution; (**b**) correlation coefficient distribution; (**c**) coefficient of variation distribution.

Below, the field flight echo data of the CSAR carried by an SRUAV are taken as an example. The system parameters are shown in Table 1. The relationship between raw data energy and the rotation angle is shown in Figure 6a, and the relationship between the raw data correlation coefficient and the rotation angle is shown in Figure 6b.

In Figure 6a, five typical raw data intensity energy points are marked in the red dashed ellipse. The peak values of the five typical correlation coefficients are marked with red dashed ellipses in Figure 6b. The five red dashed ellipses in Figure 6a correspond to the positions of the five red dashed ellipses in Figure 6b. This indicates that the variation trend in the Pearson correlation coefficient is consistent with the variation trend in raw data energy. However, it cannot distinguish the energy mutation position, and other parameters are needed to assist it. Therefore, the variation in raw data energy is characterized by the correlation coefficient of raw data. However, the correlation coefficient cannot fully characterize the anisotropy of target scattering.

In Step 1.2, the candidate sub-aperture boundary points $t_{\mathrm{imin}}$ are determined.

According to the relationship between the CSAR system resolution and the corresponding rotation angle of the sub-aperture [34], the rotation angle of the maximum sub-aperture can be approximated:

(9) $$\varphi_{\mathrm{submax}}=2\arcsin\left(\frac{c}{4f_{c}\cdot ∆\delta }\right)$$

where $\varphi_{\mathrm{submax}}$ is the rotation angle of the maximum sub-aperture; $∆\delta =c/\left(2B_{r}\right)$ is the resolution of this CSAR system, and $B_{r}$ denotes the bandwidth of the CSAR system. Using the CSAR system parameters shown in Table 1, $B_{r}=0.75\;\mathrm{GHz}$, ∆δ≈0.2 m, and $\varphi_{\mathrm{submax}}\approx 4.5{}^{\circ}$. Both the selection of candidate sub-aperture boundary points and the generation of overlapping sub-aperture schemes require some redundancy of boundary points. Therefore, $\varphi_{\mathrm{submax}}/4$ was selected as the basis for dividing the correlation coefficient $P\left(t\right)$. The width of the interval is

(10) $$L_{q}=\frac{N\cdot \varphi_{\mathrm{submax}}}{8\pi }$$

The number for one interval is

(11) $$N_{q}=\left\lfloor \frac{8\pi }{\varphi_{\mathrm{submax}}}\right\rfloor$$

where $\left\lfloor \cdot \right\rfloor$ is the floor function. $P\left(t\right)$ is divided into equal-width intervals $P_{i}\left(t\right)$, where $i=1,2,\cdots ,N_{q}$, $n=1,2,\cdots ,L_{q}$.

Find the rotation angle corresponding to the minimum value of the correlation coefficient in each interval, that is, the slow sampling time $t_{\mathrm{imin}}$:

(12) $$t_{\mathrm{imin}}=\mathrm{find}\left\{P_{i}\left(t\right)=\min\left[P_{i}\left(t\right)\right]\right\},i=1,2,\cdots ,N_{q},t=1,2,\cdots ,L_{q}$$

where $\min\left(\cdot \right)$ denotes find the minimum of $P_{i}\left(t\right)$ and $\mathrm{find}\left(\cdot \right)$ represents the operation to obtain the position $t_{\mathrm{imin}}$ of $\min\left[P_{i}\left(t\right)\right]$. Since $t_{\mathrm{imin}}$ is the position with the smallest correlation coefficient in the i interval, $t_{\mathrm{imin}}$ is the position with the weakest scattering energy or the position with a sudden change of scattering energy in this interval, both of which are the least likely positions of the strong scattering energy center. Therefore, $t_{\mathrm{imin}}$ is the candidate sub-aperture boundary point.

In Step 1.3, the sub-aperture boundary points are determined by screening the candidate sub-aperture boundary points.

The coefficient of variation $C.V_{t_{\mathrm{imin}}}$ in the energy $EN\left(t_{\mathrm{imin}}\right)$ at the boundary point of each candidate sub-aperture is calculated:

(13) $$C.V_{t_{\mathrm{imin}}}=\frac{\sigma_{t_{\mathrm{imin}}}}{M_{n_{\mathrm{imin}}}}=\frac{\sqrt{\frac{1}{5}\sum_{k=-2}^{2}{\left(EN\left(t_{\mathrm{imin}}+k\right)-M_{t_{\mathrm{imin}}}\right)}^{2}}}{\frac{1}{5}\sum_{k=-2}^{2}EN\left(t_{\mathrm{imin}}+k\right)}$$

where $\sigma_{t_{\mathrm{imin}}}$ and $M_{t_{\mathrm{imin}}}$ respectively represent the standard deviation and mean near the boundary point $t_{\mathrm{imin}}$ of the candidate sub-aperture (two points before and two points after). The coefficient of variation $C.V_{t_{\mathrm{imin}}}$ is dimensionless. $C.V_{t_{\mathrm{imin}}}$ represents the ratio between the standard deviation and the mean of the energy at five points near $t_{\mathrm{imin}}$, and reflects the discrete degree of the energy distribution relatively independently, as shown in Figure 6c. The larger the coefficient of variation, the greater the difference in the data near the boundary point of the candidate sub-aperture $t_{\mathrm{imin}}$, and the more likely it is to be the part of the edge energy fluctuation in the strong scattering energy. $t_{\mathrm{imin}}$ is easily affected by the strong scattering energy and should not be used as the boundary point of the sub-aperture. The smaller the coefficient of variation, the farther the candidate sub-aperture boundary point $t_{\mathrm{imin}}$ from the strong scattering energy. The energy changes relatively slowly near $t_{\mathrm{imin}}$, so $t_{\mathrm{imin}}$ can be the sub-aperture boundary point.

The coefficient of variation $C.V_{t_{\mathrm{imin}}}$ is compared to the threshold. If $C.V_{t_{\mathrm{imin}}}>C_{\mathrm{threshold}}$, the candidate sub-aperture boundary point is close to the strong scattering energy, and this candidate point is removed. If $C.V_{t_{\mathrm{imin}}}<C_{\mathrm{threshold}}$, the candidate sub-aperture boundary point is far away from the strong scattering energy, and this candidate point is considered as the sub-aperture boundary point $b_{\mathrm{imin}}$, $i=1,2,\cdots ,N_{\mathrm{sub}}\left(N_{\mathrm{sub}}\leq N_{q}\right)$. According to the distribution of $C.V_{t_{\mathrm{imin}}}$ for multiple groups of data, let the threshold be 20% of the maximum value of $C.V_{t_{\mathrm{imin}}}$, $C_{\mathrm{threshold}}=0.054$.

### 3.2. Automatically Generate Overlapping Sub-Aperture Schemes

In Step 2.1, the overlapping sub-aperture schemes are generated based on the above sub-aperture boundary points $b_{\mathrm{imin}}$.

**Figure 7:**
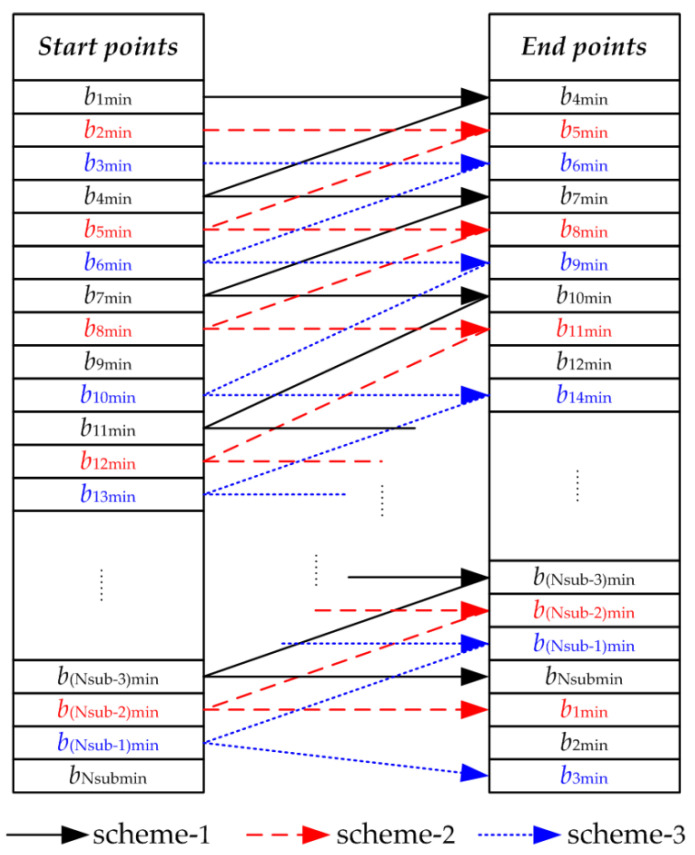
Generation of sub-aperture schemes.

The rotation angle $\varphi_{\mathrm{sub}}$ of each sub-aperture in these schemes should be as close to $\varphi_{\mathrm{submax}}$ as possible and meet $\varphi_{\mathrm{sub}}\leq \varphi_{\mathrm{submax}}$. Since $\varphi_{\mathrm{submax}}/4$ is used as the basis for the division of correlation coefficient $P\left(t\right)$, three schemes can automatically be generated according to the above conditions. The process of automatically generating sub-aperture schemes is shown in Figure 7.

In Figure 7, the solid black line, dotted red line, and blue point line, respectively, represent the generation process of the three schemes. For example, $b_{1\min}$ is taken as the start point of sub-aperture 1; the end point $b_{4\min}$ of sub-aperture 1 must meet the requirement that the rotation angle $\varphi_{\mathrm{sub}}$ of sub-aperture 1 should be as close to $\varphi_{\mathrm{submax}}$ as possible, and $\varphi_{\mathrm{sub}}\leq \varphi_{\mathrm{submax}}$. Next, $b_{4\min}$ is taken as the start point of sub-aperture 2, and the end point of sub-aperture 2 is found at the sub-aperture boundary points according to the conditions, and so on, until the full aperture CSAR data are divided. The start and end points of each sub-aperture are recorded in the scheme. Some sub-aperture boundary points may not be used during the generation of the above sub-aperture scheme. Because the CSAR trajectory is a closed circle, the endpoint of the last sub-aperture in Schemes 2 or 3 may be near the starting position of the CSAR trajectory rather than near the end.

In Step 2.2, the average sub-aperture width of each scheme is calculated:

(14) $$M_{\varphi_{\mathrm{subi}}}=\frac{1}{N_{\mathrm{subi}}}\sum_{k=1}^{N_{\mathrm{subi}}}\varphi_{\mathrm{subi}}\left(k\right)$$

where $\varphi_{\mathrm{subi}}\left(k\right)$ represents the width of each sub-aperture in the scheme $B_{i}\left(i=1,2,3\right)$; $k=1,2,\cdots ,N_{\mathrm{subi}}$ is the serial number of the sub-aperture in the scheme i; $N_{\mathrm{subi}}$ represents the number of sub-aperture in the scheme i.

Then, two schemes with larger average sub-aperture widths $M_{\varphi_{\mathrm{subi}}}$ are selected as the overlapping sub-aperture schemes $B_{m1}$ and $B_{m2}$ to ensure that the resolution of the sub-aperture images is as high as possible.

### 3.3. Sub-Aperture BP Imaging

The BP algorithm is used to image each sub-aperture under a unified grid. The overlapping sub-aperture image sequences $I_{\mathrm{sub}1}=\left[i_{s\mathrm{ub}11},i_{s\mathrm{ub}12},\cdots ,i_{1N_{s\mathrm{ub}1}}\right]$ and $I_{\mathrm{sub}2}=\left[i_{s\mathrm{ub}21},i_{s\mathrm{ub}22},\cdots ,i_{2N_{s\mathrm{ub}2}}\right]$ are obtained, where $i_{s\mathrm{ubjk}}$ is the sub-aperture image in each overlapping sub-aperture scheme. When j=1, $k=1,2,\cdots ,N_{s\mathrm{ub}1}$, and when j=2, $k=1,2,\cdots ,N_{s\mathrm{ub}2}$. The imaging grid takes the CSAR system resolution ∆δ≈0.2 m as a reference, and the minimum span of the grid is 0.2 m.

### 3.4. Sub-Aperture Image Registration Fusion

In Step 4.1, according to the scattering characteristics of the target in the observation scene $[i_{1},i_{2},\cdots ,i_{N_{s\mathrm{ub}1}+N_{s\mathrm{ub}2}}]=\left[i_{s\mathrm{ub}11},i_{s\mathrm{ub}21},\cdots ,i_{\mathrm{sub}1N_{s\mathrm{ub}1}},i_{\mathrm{sub}2N_{s\mathrm{ub}2}}\right]$, the overlapping sub-aperture image sequence is arranged according to the rotation angle corresponding to the center of the sub-aperture.

In Step 4.2, the $N_{s\mathrm{ub}1}+N_{s\mathrm{ub}2}$ sub-aperture images are divided into M parts, with each part being composed of $N_{K}$ overlapping sub-apertures and part j ($1\leq j\leq M-N_{K}$) containing the sub-aperture image sequence $\left[i_{j},i_{j+1},\cdots ,i_{j+N_{K}/2},\cdots ,i_{j+N_{K}}\right]$.

In Step 4.3, the sub-aperture image $i_{j+N_{K}/2}$ is selected as the reference sub-aperture image, and the local shape autocorrelation registration method based on normalized cross-correlation is adopted to process and record the sub-aperture image in each part as the reference sub-aperture image [40]. The cumulative results for each part are as follows:

(15) $$A_{j}=\frac{1}{N_{K}}\sum_{j}^{j+N_{K}}i_{j}\begin{array}{cc} & \left(1\leq j\leq M-N_{K}\right) \end{array}$$

In Step 4.4, after rotating the image according to the center angle of each part of the image, the final CSAR image is obtained using incoherent superposition.

## 4. Experiments and Analysis

### 4.1. Experimental Data and Methods

The X-band CSAR raw data used in this paper were obtained from the Chang’an District of Shijiazhuang by a CSAR system carried by an SRUAV; the parameters of the CSAR system are shown in Table 1.

**Figure 8:**
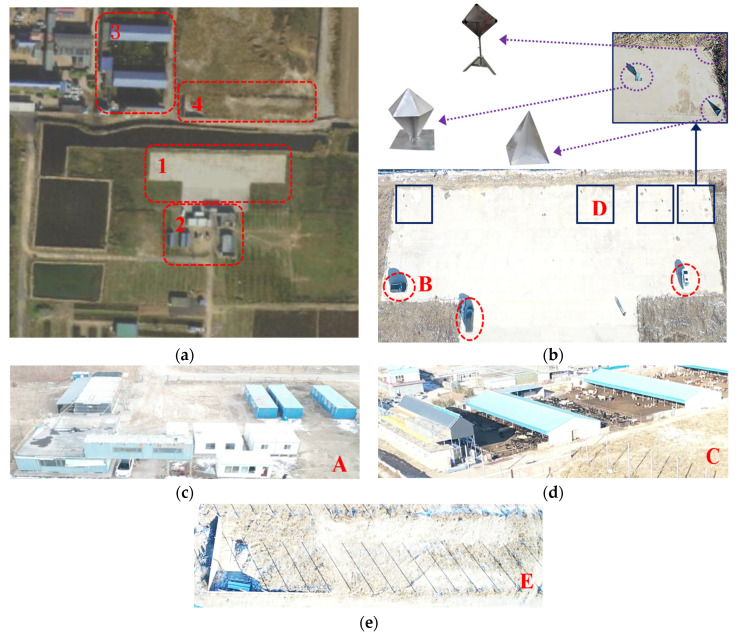
Optical photograph of the observation scene and target description; (**a**) Google Earth image of the observation scene; (**b**) optical photograph of Region 1 and target description; (**c**) optical photograph of Region 2; (**d**) optical photograph of Region 3; (**e**) optical photograph of Region 4.

The scene satellite image is shown in Figure 8a. Region 1 was a T-shaped concrete floor, as shown in Figure 8b. Three vehicles were arranged in the position circled by the red dotted ellipse, defining the vehicle on the left as target B. Four corner reflector groups were arranged in the position framed by the solid blue line box, each consisting of a tetrahedral corner reflector, a hexahedral corner reflector, and a dihedral corner reflector, defining the second corner reflector group on the left as target D. Region 2 was a group of small buildings composed of loose containers, as shown in Figure 8c, defining the small buildings as target A. Region 3 was a breeding farm, in which there were several sheds with colored steel roofs, as shown in Figure 8d, defining the colored steel roofs as target C. Region 4 comprised numerous regular and thin metal columns, each being equivalent to a total reflection target, as shown in Figure 8e, defining the metal columns as target E.

**Figure 9:**
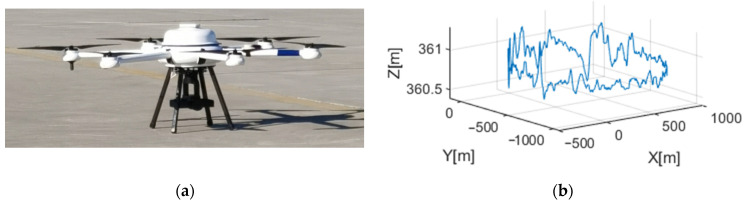
CSAR system carried by an SRUAV and its trajectory; (**a**) photograph of the CSAR system carried by the SRUAV; (**b**) flight trajectory of the CSAR system carried by the SRUAV.

The X-band CSAR system carried by an SRUAV is shown in Figure 9a, and its flight trajectory is shown in Figure 9b. During CSAR mode observation, the three-axis self-stabilizing platform of the airborne CSAR was closed, and the center direction of the antenna beam was kept perpendicular to the flight direction of the SRUAV.

Due to the low accuracy of airborne GPS and INS, the motion error compensation requirements for accurate CSAR imaging could not be met. Therefore, all subsequent imaging was performed on the original data without GPS and INS data. The CSAR original echo data comprised 180,000 sampling points along the full aperture of the circular track and 5000 sampling points in the distance. The data processing used Matlab R2019b, and the processor was a 2.5 GHz i7-6500U CPU.

### 4.2. Analysis of the Results

Based on the performance of the CSAR system on the SRUAV, the BP imaging grid was conducted uniformly; the imaging resolution was ∆δ≈0.2 m and the range was 300 m ×300 m. The CSAR full-aperture BP imaging algorithm and the equalization overlapping sub-aperture CSAR imaging algorithm were used as comparison algorithms and compared to the adaptive overlapping sub-aperture CSAR imaging algorithm proposed in this paper. The details are as follows:

Method 1: The CSAR full-aperture BP imaging algorithm.

**Figure 10:**
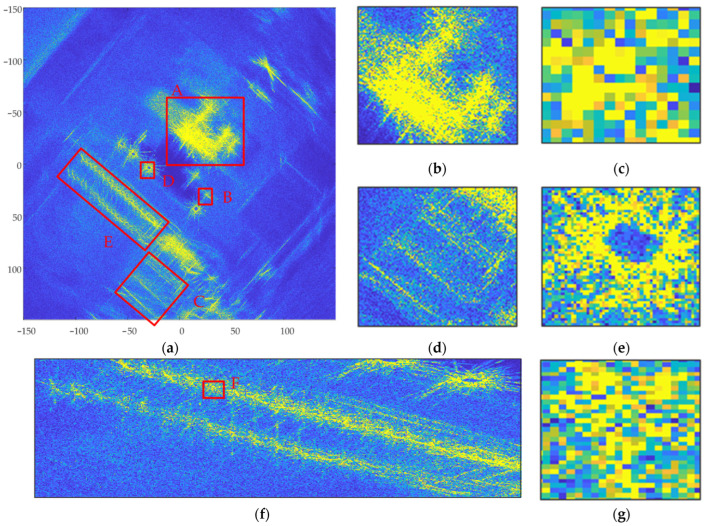
Imaging results for the CSAR full-aperture BP imaging algorithm; (**a**) complete scene CSAR image; (**b**) region A; (**c**) region B; (**d**) region C; (**e**) region D; (**f**) region E; and (**g**) region F.

The CSAR full-aperture raw data comprising 180,000 points performed 360° fully coherent BP imaging in the ground Cartesian coordinate system; the results are shown in Figure 10.

Method 2: The equalization overlapping sub-aperture CSAR imaging algorithm.

**Figure 11:**
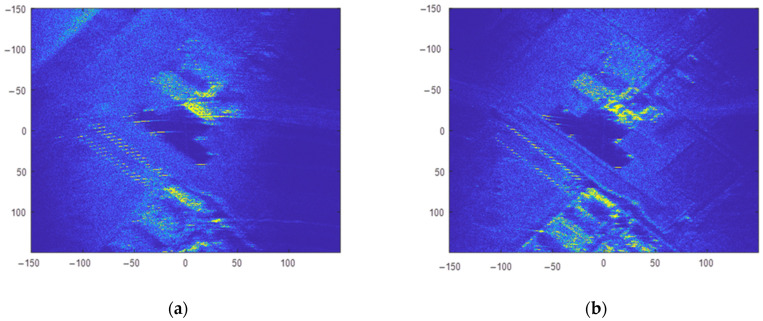
Sub-aperture image acquired using the equalization overlapping sub-aperture CSAR imaging algorithm; (**a**) sub-aperture 1 image; (**b**) sub-aperture 80 image.

**Figure 12:**
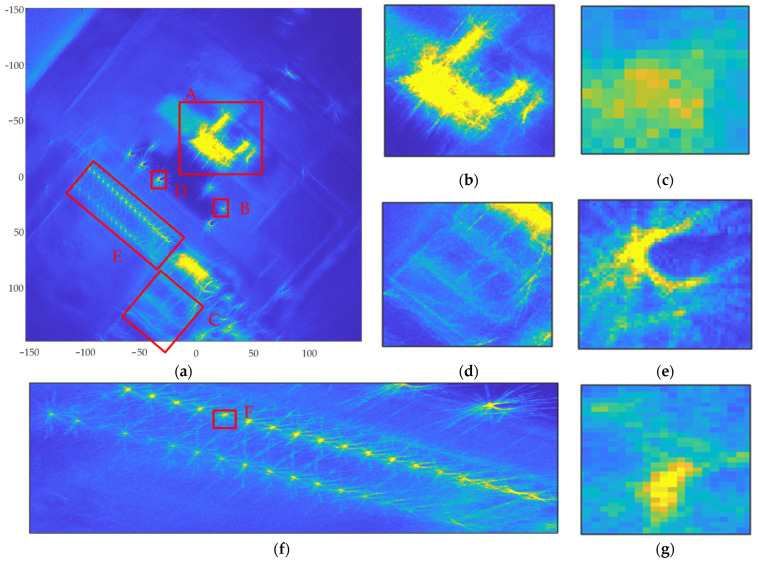
Imaging results for the equalization overlapping sub-aperture CSAR imaging algorithm; (**a**) complete scene CSAR image; (**b**) region A; (**c**) region B; (**d**) region C; (**e**) region D; (**f**) region E; and (**g**) region F.

First, the CSAR raw data were divided into 160 overlapping sub-apertures with an equal rotation angle of 4.5°. Each sub-aperture contained 2250 slow-time sampling points, and the overlap ratio of sub-apertures was 50%. The BP algorithm was then used to focus the raw data for each sub-aperture in the ground Cartesian coordinate system. The images for sub-aperture 1 and sub-aperture 80 are shown in Figure 11. The resolution of sub-aperture images was low, and the target information contained in the sub-aperture images with different rotation angles was different. Finally, all sub-aperture images were incoherently superimposed; the results are shown in Figure 12.

Method 3: The adaptive overlapping sub-aperture CSAR imaging algorithm.

**Figure 13:**
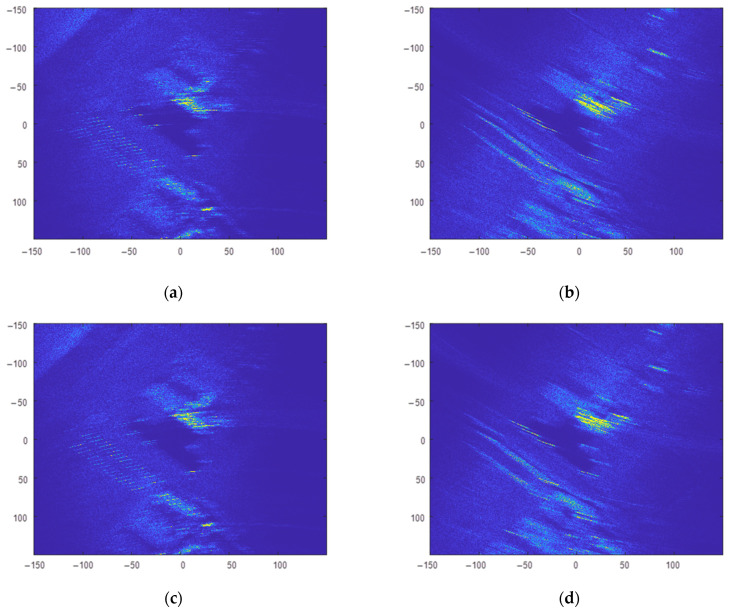
Sub-aperture image of the adaptive overlapping sub-aperture CSAR imaging algorithm; (**a**) Scheme 1, sub-aperture 1 image; (**b**) Scheme 1, sub-aperture 40 image; (**c**) Scheme 2, sub-aperture 1 image; and (**d**) Scheme 2, sub-aperture 40 image.

**Figure 14:**
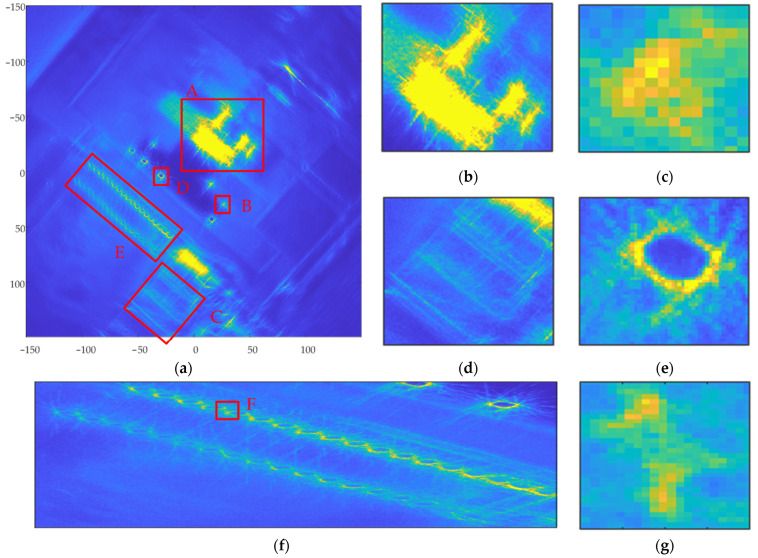
Imaging results acquired using the adaptive overlapping sub-aperture CSAR imaging algorithm; (**a**) whole scene CSAR image; (**b**) local magnification of region A; (**c**) local magnification of region B; (**d**) local magnification of region C; (**e**) local magnification of region D; (**f**) local magnification of region E; and (**g**) local magnification of region F.

The processing flow of the proposed imaging algorithm in this paper is shown in Figure 5. First, 301 sub-aperture boundary points were determined using variation system analysis of the echo coherence coefficient and energy function, which were smaller than the number of candidate sub-aperture boundary points $N_{q}=\left\lfloor \frac{8\pi }{\varphi_{\mathrm{submax}}}\right\rfloor$. Next, three overlapping sub-aperture partitioning schemes were automatically generated, the average length of sub-aperture in each scheme being 1942.6, 1844.0, and 1853.2, respectively. Schemes 1 and 3 were selected as overlapping sub-aperture partitioning schemes based on the optimal resolution. Scheme 1 contained 81 sub-apertures, and Scheme 2 contained 82 sub-apertures. The boundary points of the two schemes were not repeated. The BP algorithm was used to focus the raw data for each sub-aperture in the ground Cartesian coordinate system. The images for sub-aperture 1 and sub-aperture 40 are shown in Figure 13. Schemes 1 and 3 were combined to form 163 overlapping sub-apertures. For the convenience of illustration, the sub-apertures in Schemes 1 and 3 are arranged independently. In Scheme 1, sub-aperture 1 started at 665 and contained 1712 slow-time sampling points; sub-aperture 40 started at 75,195 and contained 2018 slow-time sampling points. In Scheme 2, sub-aperture 1 started at 1862 and contained 1545 slow-time sampling points; sub-aperture 40 started at 76,108 and contained 2052 slow-time sampling points. The starting points of the sub-apertures acquired using the two schemes were different, but the lengths of the sub-apertures were close, so the resolution difference in the sub-aperture images is not obvious; however, the target information contained in the images of the same sub-aperture for the different schemes is different, and the image focusing result is better than those in Method 2 sub-aperture images. Finally, sub-aperture image registration and incoherent superposition were used to generate the final CSAR image, as shown in Figure 14.

In Figure 10, Figure 12 and Figure 14, (a) represents the panoramic view of the observation scene, with an imaging range of 300 m ×300 m; and (b) to (g) represent local enlargements of areas A, B, C, D, E, and F marked in (a), respectively.

The full-scene CSAR images acquired using these three methods are shown in Figure 10a, Figure 12a and Figure 14a. In Figure 10a, the full scene CSAR image acquired using the CSAR full-aperture coherent imaging method exhibits serious defocus. Compared with Figure 10a, the focusing effect in the complete scene CSAR image acquired using the evenly overlapping sub-aperture CSAR imaging method is significantly improved, as shown in Figure 12a. The result of the adaptive overlapping sub-aperture CSAR imaging method shown in Figure 14a effectively improved the focusing effect of weakly scattered energy targets, such as the breeding farm and roads, on the premise that the focusing effect was the same as that shown in Figure 12a.

Image entropy was used as an index to quantitatively measure the images. The expression for calculating image entropy is as follows:

(16) $$\left\{\begin{array}{l} S=-\sum_{i=1}^{m}\sum_{j=1}^{n}\rho_{ij}\log\rho_{ij}\\ \rho_{ij}=\frac{{\left|x_{ij}\right|}^{2}}{C}\\ C=\sum_{i=1}^{m}\sum_{j=1}^{n}{\left|x_{ij}\right|}^{2}n \end{array}\right.$$

where $\rho_{ij}$ represents the ratio of the energy of a pixel $x_{ij}$ in the image to the total energy of the image C.

The image entropy calculated for the three imaging algorithms is shown in Table 2. Compared with Method 1, the scene image entropies for Methods 2 and 3 increased significantly by 65.92% and 66.77%, respectively. The full scene image entropy for Method 3 increased by 0.5% compared with Method 2.

According to the scattering energy characteristics of the targets in the scene, these targets can be divided into isotropic targets (for example, the metal columns and the corner reflectors) and anisotropic targets (for example, other man-made targets in the scene). According to the intensity of scattering energy, the anisotropic targets can be divided into strong scattering energy targets (for example, the loose containers and cars) and weak scattering energy targets (for example, the breeding farm). The following analysis was performed according to three categories: anisotropic targets with strong scattering energy, anisotropic targets with weak scattering energy, and isotropic targets.

#### 4.2.1. Anisotropic Targets with Strong Scattering Energy

**Figure 15:**
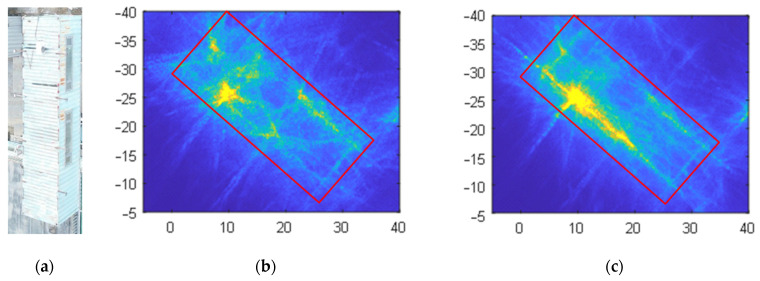
Images of the container; (**a**) optical photograph; (**b**) Method 2 image; (**c**) Method 3 image.

The CSAR images of the loose containers in Figure 8 correspond to region A in Figure 10a, Figure 12a and Figure 14a, respectively, and are shown in Figure 10b, Figure 12b and Figure 14b after local amplification. Compared with Figure 10b, the focusing effects of Figure 12b and Figure 14b are significantly improved, and the contour of the containers in Figure 14b is clearer, as shown in Figure 15.

The CSAR images of Vehicle B in Figure 8 correspond to region B in Figure 10a, Figure 12a and Figure 14a, respectively. After local magnification, the same car is shown in Figure 10c, Figure 12c and Figure 14c. In Figure 10c, the contour of the car is not at all distinguishable; Figure 12c only shows the contour of the car faintly, but it is indistinguishable from the background; the contour of the car in Figure 14c is clearer.

The image entropies of two anisotropic target regions with strong scattering energy were analyzed, as shown in the corresponding rows in Table 2. Compared with Method 1 imaging results, Method 2 image entropy increased by 30.10% (A region) and 17.03% (B region), respectively. Method 3 image entropy increased by 31.85% (A region) and 17.91% (B region), respectively; Method 2 and Method 3 image entropy, therefore, increased significantly compared with that of Method 1. Method 3 exhibits some improvement on Method 2, with the image entropy of region A increasing by 1.34% and the image entropy of region B increasing by 0.76%.

#### 4.2.2. Anisotropic Target with Weak Scattering Energy

The CSAR images of the breeding farm in Figure 6 correspond to region C in Figure 10a, Figure 12a and Figure 14a, respectively, and are shown in Figure 10d, Figure 12d and Figure 14d after local amplification. In Figure 10d, there is severe defocus and low SNR. In Figure 12d, the contours of the sheds are faintly visible, but the edges of the contours are defocused. Figure 14d exhibits good focus with better contour details.

The image entropy of the anisotropic target region with weakly scattered energy was analyzed, as shown in the corresponding rows in Table 2. Compared with the imaging results for Method 1, the image entropies for Methods 2 and 3 increased by 12.73% and 15.24%, respectively. Compared with Method 2, the image entropy for Method 3 increased by 1.33%. Among the three imaging algorithms, Method 3 produced the highest image entropy and the best imaging effect.

#### 4.2.3. Isotropic Targets

The CSAR images of corner reflector group D in Figure 8 correspond to region D in Figure 10a, Figure 12a and Figure 14a, respectively, and are shown in Figure 10e, Figure 12e and Figure 14e after local amplification. The positions of the three corner reflectors in Figure 10e could not be distinguished at all. Figure 12e shows that the tetrahedral corner reflector was located, but its energy was too strong, completely drowning the other two corner reflectors, so the position of the other two corner reflectors could not be determined. Figure 14e reflects the triangular arrangement of the three corner reflectors.

The CSAR images of the regular and thin metal columns in Figure 8 correspond to region E in Figure 10a, Figure 12a and Figure 14a, respectively, and are shown in Figure 10f, Figure 12f and Figure 14f after local magnification. Compared with Figure 10f, the focusing effects seen in Figure 12f and Figure 14f significantly improved. A metal column in region E was selected to be enlarged separately, as shown in Figure 10g, Figure 12g and Figure 14g. In Figure 12g, the position of this metal column was realized, but in Figure 14g, in addition to the position of this metal column, the elongated shape can also be seen.

The image entropies of the three isotropic target regions, D, E, and F, were analyzed, as shown in the corresponding rows in Table 2. Compared with the imaging results acquired by Method 1, Method 2 image entropy increased by 2.27% (D region), 7.54% (E region), and 4.83% (F region), and Method 3 image entropy increased by 13.65% (D region), 17.08% (E region), and 8.37% (F region), respectively. Methods 2 and 3 image entropies were significantly higher than those acquired in Method 1. Method 3 image entropy significantly improved on that of Method 2. The image entropy for region D increased by 11.12%, that for region E by 8.87%, and that for region F by 3.38%.

In summary, the proposed method achieved focused images using CSAR raw data without GPS and INS; in addition, the imaging effect improved compared with the CSAR full-aperture coherent imaging method and the evenly overlapping sub-aperture CSAR imaging method. For anisotropic targets with weak scattering energy, the proposed method can significantly improve the details of the target. For anisotropic and isotropic targets with strong scattering energy, the focusing effect can be improved, the target contour can be improved, and the mutual influence of the strong scattering targets can be weakened.

## 5. Conclusions

Analysis of CSAR time-domain imaging methods based on sub-aperture found that existing airborne methods only consider phase compensation and ignore the influence of target scattering on raw data amplitude in the observation scene. For CSAR systems carried by an SRUAV, compared to the distance between the target and the antenna, the size of the observation scene cannot be ignored. Phase compensation causes the strong energy sidelobe to subdue the weak energy main lobe, low SNR, as well as serious defocus in the image. Therefore, the effect of anisotropy of target scattering energy on the raw data amplitude must be considered. Based on this, we propose an adaptive overlapping sub-aperture CSAR imaging method. First, the sub-aperture boundary points are determined based on the joint analysis of the correlation coefficient and coefficient of variation; next, the overlapping sub-aperture schemes are generated and used to divide the raw data into sub-aperture data; the BP algorithm is then used to complete the focused imaging of each sub-aperture; finally, all the sub-aperture images are generated by registration fusion. The proposed method ensures that the strong scattering energy is completely located in one sub-aperture, and the width of the sub-aperture is as close as possible to the theoretical maximum sub-aperture width. This can effectively reduce the influence of the strong energy sidelobe, preserve the detailed features of the targets, and improve the CSAR imaging effect. The results of X-band CSAR outfield flight raw data acquired by an SRUAV without INS and GPS demonstrate the correctness and effectiveness of the proposed method. The complete scene image entropy of the adaptive overlapping sub-aperture CSAR imaging algorithm is 66.77% higher than that of the CSAR full-aperture BP imaging algorithm, and the imaging effect significantly improved. Compared to the equalization overlapping sub-aperture CSAR imaging algorithm, the imaging effect improved by 0.5%, which is a slight improvement. For anisotropic targets with strong scattering energy, the image entropy is the most obvious improvement (31.85%) compared with the full-aperture BP imaging algorithm, improving the focusing effect of targets while weakening the mutual influence of targets with strong scattering energy. For anisotropic targets with weak scattering energy, the image details improved significantly compared with the equipartition overlapping sub-aperture imaging algorithm. For isotropic targets, the image entropy improved by 11.12% compared with the equipartition overlapping sub-aperture imaging algorithm, obviously improving the focusing effect and the target contour.

## Figures and Tables

**Table 1:** CSAR system parameters.

Parameter	Value	Parameter	Value
waveband	X	flight radius	600 m
bandwidth	0.75 GHz	flight height	300 m
PRF	333.3 Hz	velocity	7 m/s
slant range	500~1000 m		

**Table 2:** Image entropy and relative change statistics.

Scene	Method 1	Method 2		Method 3		
	$\mathrm{Entropy}\;E_{1}$	$\mathrm{Entropy}\;E_{2}$	$\frac{E_{2}-E_{1}}{E_{1}}\times 100\%$	$\mathrm{Entropy}\;E_{3}$	$\frac{E_{3}-E_{1}}{E_{1}}\times 100\%$	$\frac{E_{3}-E_{2}}{E_{2}}\times 100\%$
full scene	2.2576	3.7459	65.92%	3.7650	66.77%	0.5%
region A	4.3104	5.6078	30.10%	5.6831	31.85%	1.34%
region B	5.8517	6.8480	17.03%	6.8998	17.91%	0.76%
region C	5.7254	6.5113	12.73%	6.5981	15.24%	1.33%
region D	5.8484	5.9813	2.27%	6.6466	13.65%	11.12%
region E	5.2842	5.6828	7.54%	6.1866	17.08%	8.87%
region F	6.5236	6.8384	4.83%	7.0698	8.37%	3.38%

## Data Availability

The data that support the findings of this study are available on request from the corresponding author, [Ma Y.], upon reasonable request.
